# Atomic Force Microscopy Investigation of DNA Extracted from the Vegetative Forms and the Viable but Nonculturable Forms of *Mycoplasma gallisepticum* S6

**DOI:** 10.1100/tsw.2008.143

**Published:** 2008-11-02

**Authors:** Olga A. Chernova, Maxim V. Trushin, Alexey A. Mouzykantov, Vladislav M. Chernov

**Affiliations:** Kazan Institute of Biochemistry and Biophysics, Russian Academy of Sciences, Kazan, Russian Federation

**Keywords:** mycoplasma, atomic force microscopy, DNA, viable but nonculturable (VBNC) forms, vegetative forms

## Abstract

Recent studies show that mycoplasmas have various programs of life. This means that changes in morphology and genome expression may occur once the environment of these microorganisms becomes extremely altered. In this article, we report on changes in the DNA molecule obtained from the vegetative forms and the viable but nonculturable (VBNC) forms of *Mycoplasma gallisepticum* S6. Atomic force microscopy studies show that the above-mentioned forms of the mycoplasma have different values of DNA parameters (height: 0.461 ± 0.141 and 0.236 ± 0.069 nm; width: 2.221 ± 0.286 and 1.291 ± 0.705 nm for the vegetative and the VBNC forms, respectively). We suppose that the observed phenomenon may be connected with the process of adaptation of these bacteria to severe environments.

## INTRODUCTION

Mycoplasmas (class *Mollicutes*) are unique bacteria that are characterized by a small cell size, a reduced genome, and the absence of a cell wall[1]. Despite their seeming simplicity, little is known about the precise mechanisms that possess the virulence and pathogenicity of mycoplasmas. Recently, the principal ability to change morphologic, genetic, and biochemical features during stress conditions was reported for mycoplasma cells[2,3] as well as for other bacteria. These changes may include a reduction of the cell size, a disappearance of cell polarity, and an alteration of expression of some genes[4,5]. These modified cells were referred to as the "viable but nonculturable" (VBNC) forms.

We found that changes in DNA structure take part during adaptation of *Mycoplasma gallisepticum* S6 to stress conditions. In the VBNC forms of this mycoplasma, we detected the nucleotide sequence (582 bp) with two open-reading frames that is not registered in the vegetative forms of *M. gallisepticum* S6.

Because of the revealed DNA modifications, it is reasonable to expect some global changes in the structure of DNA, particularly in its secondary structure. Therefore, in the present study, we intend to compare the height and other DNA characteristics of the DNA molecules extracted from the vegetative forms and the VBNC forms of *M. gallisepticum* S6 as measured by atomic force microscopy (AFM).

## MATERIALS AND METHODS

The *M. gallisepticum* S6 strain was obtained from the N.F. Gamalei Research Institute of Epidemiology and Microbiology (Moscow, Russia).

*M. gallisepticum* S6 cells grown in the liquid-modified Edward's nutrient medium for 3 days and grown in deficient medium for 26 weeks (cells adapted to unfavorable growth conditions) were used to compare their DNA properties. The liquid-modified Edward's nutrient medium[3] consisted of the following components (in %): tryptose, 2; NaCl, 0.5; KCl, 0.13; Tris basic, 0.3; serum of horse blood, 10; yeast extract, 5; glucose, 1. It also contained penicillin, 1000 U/mL, and phenol red, 0.3 mL 1%-solution. In the case of the deficient medium, glucose, yeast extract, and serum of horse blood were excluded.

For AFM studies, DNA was extracted from *M. gallisepticum* S6 cells grown in the full-growth medium and in the deficient one (the vegetative forms and the VBNC forms, respectively). To extract DNA, the following procedures were applied. Mycoplasma cells were centrifuged at 9000*g* for 20 min. The pellet was resuspended in TES buffer (Tris-HCl 10 m*M*, EDTA 1 m*M*, NaCl 100 m*M*), SDS (1–2%) was added to the solution, shaken, and stored at 37°C. An equal volume of aqueous phenol-chloroform mixture (1:1) was added to the obtained cell lysate. Aqueous phase was separated by centrifugation and the second extraction with chloroform-isoamyl alcohol mixture (24:1) was performed. DNA was precipitated with 2.5 volume of ethanol (96%). The pellet was dissolved in TE buffer (Tris-HCl 10 m*M*, Na-EDTA 1 m*M*, pH 8.0). For additional purification, the solution was incubated with RNAse (20 μg/mL, Serva, Germany) for 30 min at 37°C and then with proteinase K (50 μg/mL, Sigma, USA) for 30 min at 37°C. After the enzymatic treatment, DNA was again extracted with the chloroform-isoamyl alcohol mixture and precipitated with 2.5 volume of ethanol (96%). DNA concentration was detected in agarose gel electrophoresis using standard DNA samples (Fermentas, Lithuania).

A buffer containing 5 m*M* $MgCl_2$ and 10 m*M* Tris was used to dilute DNA samples to special concentrations (0.15–0.5 ng/μL). The upper layer of mica (Advanced Technologies Center, Moscow, Russia) was eliminated with scotch and DNA samples (3 μL) were placed onto the mica for 1 min. Then the mica with DNA was rinsed twice with redistilled water and, after each rinsing, it was dried with the pressurized air. Images were acquired in air by a Solver P47H atomic force microscope (NT-MDT, Moscow, Russia) operating in the tapping mode using fpN11S cantilevers (r = 10 nm, Advanced Technologies Center, Moscow, Russia). The height, Mag (signal from lock-in amplifier), RMS (signal from RMS detector), and Phase (signal from the phase detector) were performed with the Nova 1.0.26 RC1 software (NT-MDT, Moscow, Russia).

Data are given as mean ± SD. The Kolmogorov-Smirnov test was applied to test whether our data yielded a normal distribution. Significant differences in the results were evaluated by applying Student's *t* test. Calculations were made using the Origin 8.0 software for Windows.

## RESULTS

Dozens of DNA samples prepared as described above have been analyzed by AFM in the tapping mode in air. In many cases, the substrate appeared to be uniform with DNA molecules adsorbed onto the surface of the substrate. Rarely, however, hills and holes appeared on the surface of a mica (Fig. 1); we consider that this kind of defect can be explained by nonhomogenous distribution of the ions dissolved in the buffer containing the DNA molecules.

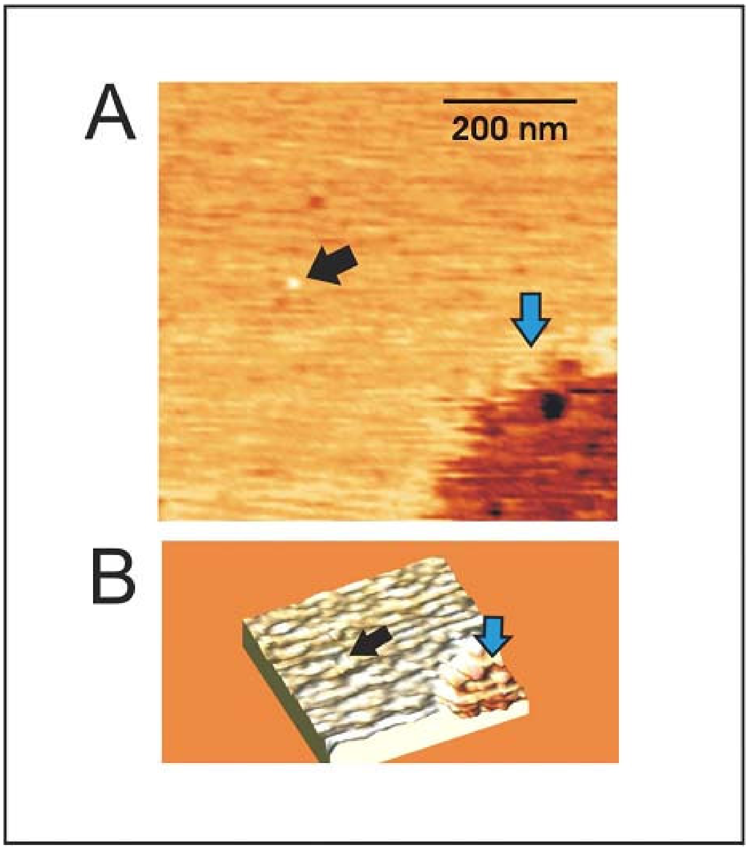

**FIGURE 1.** The substrate surface of a mica (the area without the DNA molecules, 0.6 μm$^2$). Hills and holes are indicated by black and blue arrows, respectively. (A) 2D image, the hole zone is in the right bottom; (B) 3D image.

Fig. 2 presents 2D images of DNA obtained from the vegetative forms of *M. gallisepticum* S6. In many cases, the DNA molecules were folded into compact shapes (black arrows). The general sizes of the DNA globules range between 200 and 400 nm (in diameter) and about 100 nm in height. However, smaller DNA globules were also registered (Fig. 2A,B). Also, there were DNA molecules with untwining parts (Fig. 2, green arrows) and DNAs in unstressed form (Fig. 2, red arrows). 2D images of DNA extracted from the VBNC forms of *M. gallisepticum* S6 are presented in Fig. 3. The individual DNA molecules (A–C) as well as the sites of DNA entwinement (D,E) can be seen in the figure. The differences are dictated by concentration of the molecules (0.15 and 0.45 ng/μL, respectively).

Table 1 presents data on the calculation of DNA height and width (full width at half-maximum height). Interestingly, the height of the DNA molecules taken from the VBNC forms of the mycoplasma was significantly less: 0.236 ± 0.069 nm. Similarly, the direct observation gave us the following values of DNA widths: 15.547 ± 2.002 and 9.037 ± 935 nm for the vegetative forms and the VBNC forms of *M. gallisepticum* S6, respectively. After the compilation adjustment, the sizes of DNA widths became less. We also calculated DNA widths according to formula suggested by Margeat and coworkers[6]:

$$S = 2x(R \text{ x } D + D^2/4)^{1/2}$$

where $R$ is the tip radius; $D$ is the measured height of the DNA molecule studied, S is DNA width at the half-maximum seen on the section of the DNA molecule. The performed statistical analysis (Table 2) showed significant differences between values calculated for the vegetative forms and the VBNC forms of the mycoplasma under study.

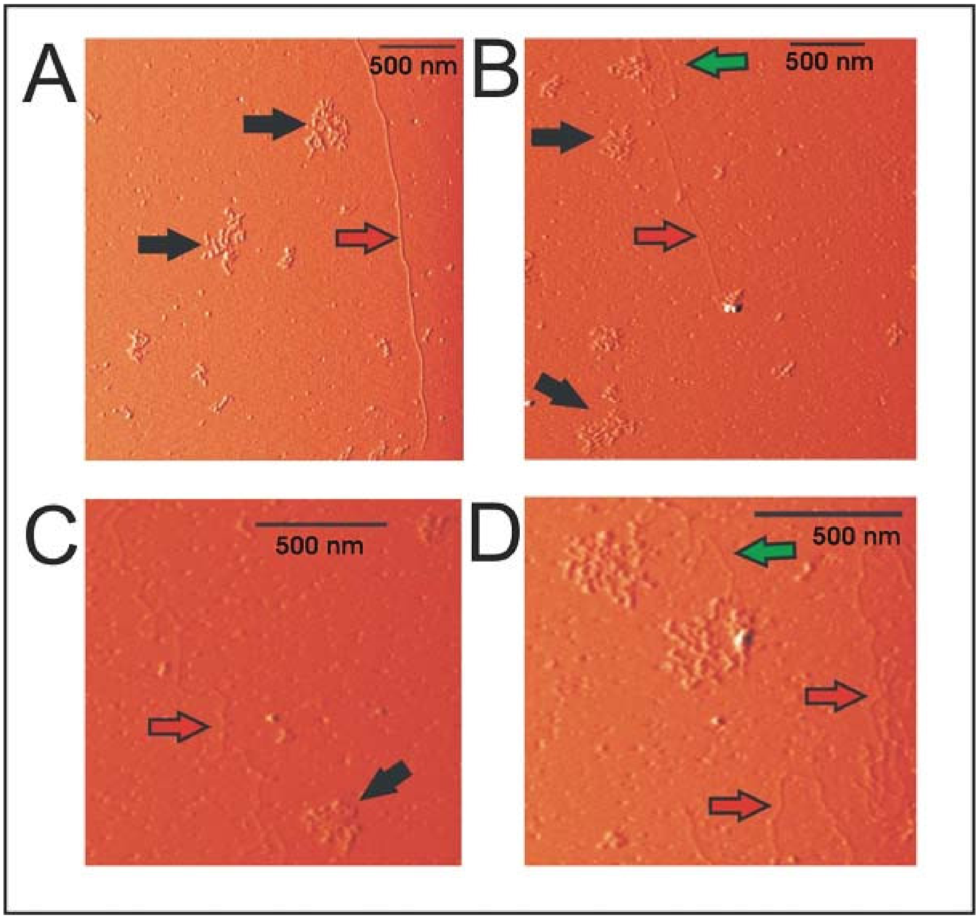

**FIGURE 2.** Noncontact tapping mode AFM of DNA obtained from the vegetative forms of *M. gallisepticum* S6 in air. (A–D) 2D images recorded from the different areas of scanning. The black arrow, the DNA molecules fold into compact shapes; green arrow, DNA in hemicompact form; red arrow, DNA in unstressed form.

## DISCUSSION

Imaging of individual biological polymers by AFM has been used productively to reveal details of their structure in a number of cases[7]. In this article, we describe for the first time the use of AFM to probe the organization of the genetic apparatus of *M. gallisepticum* S6. We have examined the topology of the DNA molecules obtained from the vegetative forms and the VBNC forms of mycoplasma. The uniformity of the substrate used allowed us to achieve 2D images of the DNA molecules with high resolution. The detection of the DNA molecules folded into compact shapes (Fig. 2, black arrows) allowed us to account for the stability of the complexes. The similar compact shapes were reported for the single-stranded ϕX-174 DNA molecules[8]. However, the sizes of the DNA globules obtained from the vegetative forms of *M. gallisepticum* S6 were greater (at least twofold) than those from ϕX-174; the variations can probably be explained by the differences in the genome size of two microorganisms. However, the nature of these compact shapes needs the further investigation.

With the use of the special software processing, AFM images of the DNA molecules obtained from the VBNC forms of *M. gallisepticum* S6 were analyzed. A few remarks can be made from the analysis. While the elevations upon substrate (indicated by black arrows in Fig. 3D,E) can be explained by DNA tangles, it is not possible to say the same about single molecules of DNA (Fig. 3A–C). It is reasonable to suggest that the sites of elevations (indicated by yellow arrows in Fig. 3A–C) present areas of DNA-Mg

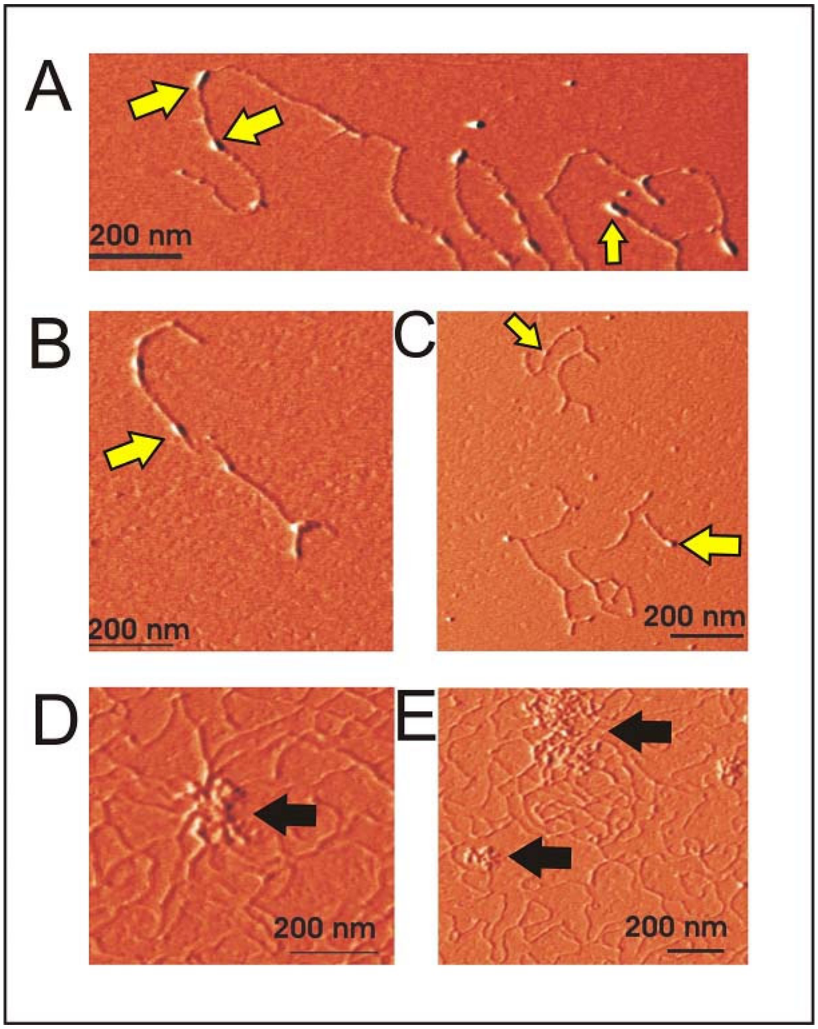

**FIGURE 3.** AFM 2D images of DNA obtained from the VBNC forms of *M. gallisepticum* S6 in air. (A–C) and (D,E) DNAs in concentration 0.15 and 0.45 ng/µL, respectively. The yellow arrows, the sites of the possible binding DNA with Mg conglomerates; black arrows, the sites of DNA entwinement.

**TABLE 1**
**Measured Height and Width of the DNA Molecules at Low Humidity (about 30%)**

| DNA Parameter (nm) | | Vegetative Forms | VBNC Forms |
|---|---|---|---|
| Height | | 0.461 ± 0.141 | 0.236 ± 0.069 |
| Width | (1) | 15.547 ± 2.002 | 9.037 ± 4.935 |
| | (2) | 2.221 ± 0.286 | 1.291 ± 0.705 |
| | (3) | 4.318 | 3.082 |

*Note*: (1) and (2) DNA width without and with the compilation adjustment (performed with the Nova 1.0.26 RC1 software), respectively. (3) DNA width calculated according to formula by Margeat et al.[6].

**TABLE 2**
**Values of Kolmogorov-Smirnov (K-S) Test for Goodness of Fit to Normal Distribution and Student's *t* Test for DNA Parameters**

| Type of *M. gallisepticum* S6 Cells | DNA Height | | DNA Width | |
|---|---|---|---|---|
| | Statistical Criterion | | Statistical Criterion | |
| | K-S | *t* | K-S | *t* |
| VBNC forms | 0.17953 | 4,76681 | 0.20161 | 4,26639 |
| Vegetative forms | 0.15851 | | 0.28201 | |

*Note*: *t* presents values of Student's *t* test for comparisons between the corresponding DNA parameters (height and width) of the VBNC forms and the vegetative forms of *M. gallisepticum* S6.

conglomerates. Their average height is 0.57 ± 0.24 nm. Because the average size of a single protein of 10 ± 100 kDa is about ~5 nm[9], it is difficult to think that these elevations were mediated by interaction of DNA with proteins. The performed deproteinization of our samples confirms this conclusion.

The most prominent changes between the DNA molecules extracted from the vegetative forms and the VBNC forms of *M. gallisepticum* S6 were detected while analyzing their height and width. The measured height of the DNA molecules extracted from the vegetative forms of *M. gallisepticum* S6 coincided with the well-accepted value for the height of the DNA as measured by AFM[10]. Concerning the DNA height measured for mycoplasma VBNC forms, we have to note that we controlled the level of humidity with the use of a hygrometer (the dependence of the DNA height on humidity in the environment is a well-known fact[11]) and the pressure of the cantilever was the same. Thus, the differences in height cannot be explained by the deformation of the DNA molecules due to these two factors. Because the technique of preparation of DNA samples was uniform for variants with the vegetative forms and the VBNC forms of *M. gallisepticum* S6 (the concentration of $MgCl_2$ was the same), the analogous conclusion can be made regarding the DNA width: the observed differences are most probably attributed to biological features of DNA obtained from the VBNC forms of the mycoplasma. The principal ability for the existence of DNA of less width (about 1–1.5 nm) (and, correspondingly, diameter) was previously reported in early X-ray diffraction studies[12,13]. It is worth noting, however, that the compilation adjustment may provoke some errors due to drawbacks in mathematical algorithm and the real radius of cantilevers may differ from the stated value. Therefore, the use of ultrasharp cantilevers is desirable for the precise measurements in AFM.

Thus, we revealed differences in DNA sizes obtained from the vegetative forms and the VBNC forms of *M. gallisepticum* S6. Very likely, the reduced sizes of DNA from the VBNC forms of the bacterium are connected with the phenomenon of nanotransformation of mycoplasmas. To test whether these events may occur in other mycoplasma species, further studies are needed.

## ACKNOWLEDGMENTS

We thank Dr. Olga A. Konovalova and Mr. Dmitry Nalimov (both from Kazan State University) for valuable help during work on the atomic force microscope. Also, we are grateful to Drs. Igor V. Yaminsky (Moscow State University), C.S. Delmonte (Open University, U.K. [Retired]), Yuri L. Lyubchenko (University of Nebraska Medical Center), Fernando Moreno-Herrero (Instituto Catalán de Nanotecnología), and Helen Hansma (University of California in Santa Barbara) for useful discussions. We thank Russian Fund for Basic Research (Project #08-04-01047a) for supporting this work.
